# Closed Genome Sequence of an Environmental Aeromonas veronii Strain from California, United States, with an IncA/C Plasmid Carrying an Extended-Spectrum β-Lactamase Gene, *bla*_VEB-3_

**DOI:** 10.1128/mra.01033-21

**Published:** 2022-02-23

**Authors:** Karissa G. Lovero, Luis Mota-Bravo

**Affiliations:** a Department of Ecology and Evolutionary Biology, University of California, Irvine, Irvine, California, USA; University of Maryland School of Medicine

## Abstract

We describe the extended-spectrum β-lactamase *bla*_VEB-3_ gene found in an IncA/C plasmid in Aeromonas veronii strain SW3814, which was collected from a freshwater lake in southern California, United States.

## ANNOUNCEMENT

*Aeromonas* bacteria are common in aquatic environments and may serve as reservoirs of antibiotic resistance genes (ARGs) (1–3). These ARGs are often associated with mobile genetic elements (MGEs) (4), which may facilitate their transfer to bacteria of clinical significance (5–7).

**FIG 1:**
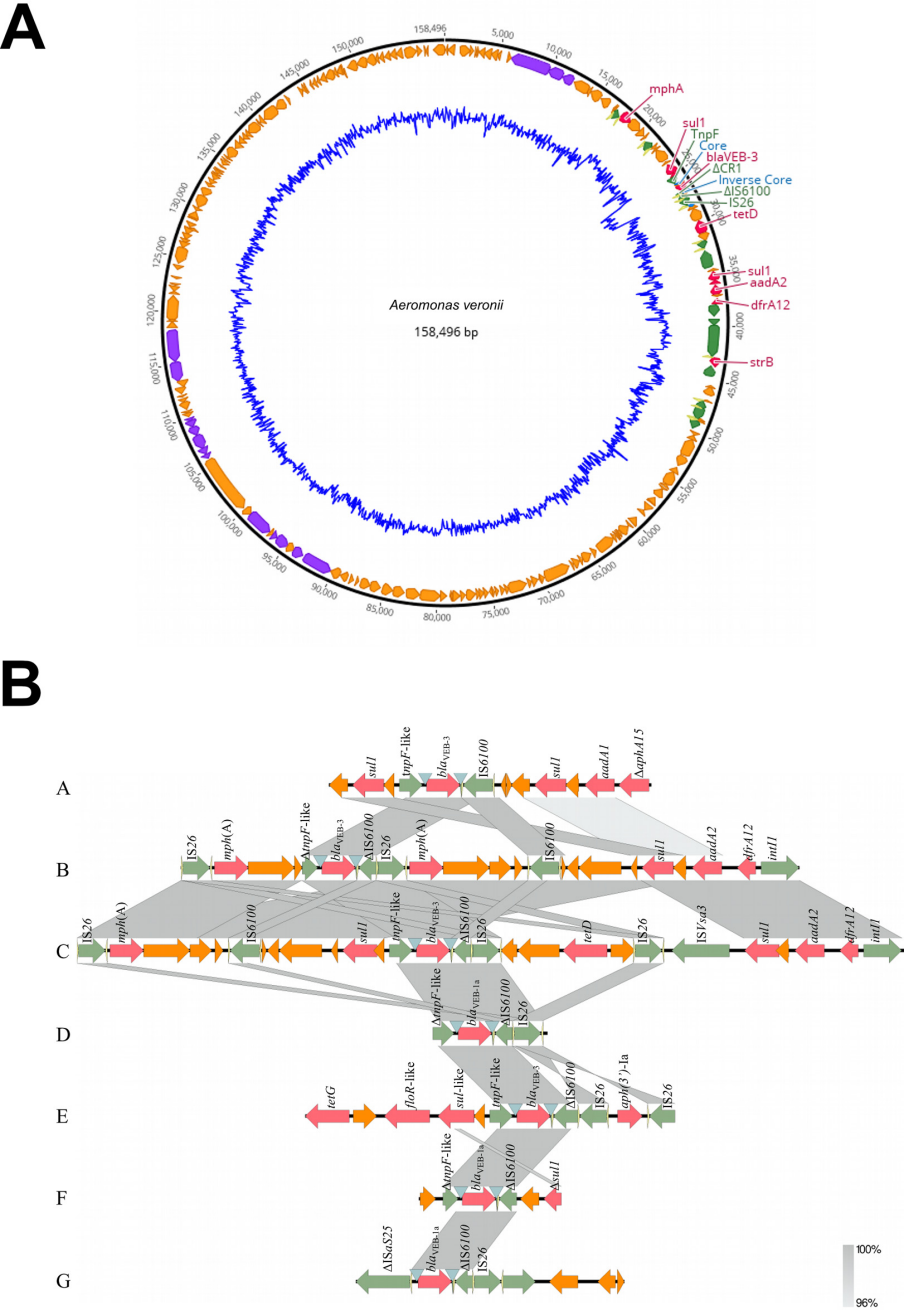
(A) Genetic map of p158496, displayed in the outermost ring. Open reading frames are represented by arrows in the direction of transcription; they are color coded according to their putative functions, as follows: purple, conjugation machinery; red, ARGs; green, MGEs; yellow, inverted repeats; orange, all other coding sequences. The inner blue ring displays the GC content of the plasmid. Genes of interest are labeled. This figure was created using Geneious v11.1.5. (B) Schematic representation of isolates from the NCBI GenBank database containing the *bla*_VEB_ gene with regions homologous to those found in p158496. Open reading frames are represented by arrows in the direction of transcription; they are color coded according to their putative functions, as follows: red, ARGs; green, MGEs; yellow, inverted repeats; orange, all other coding sequences. Inverted blue triangles represent the recombination core and inverse core sites. The identity between adjacent sequences is shown as gray shading. Δ indicates the truncation of a gene. Descriptions of isolates A through G are presented in Table 1. This figure was created using EasyFig.

Aeromonas veronii strain SW3814 was collected from a freshwater lake in southern California (English Springs Park [33.9951N, 117.756W]). Water was filtered with 0.45-μm filters (GN-6; Pall) and placed on CHROMagar orientation medium (CHROMagar, Paris, France) containing 4 μg/mL cefotaxime (Sigma-Aldrich, St. Louis, MO). A purple isolate, labeled SW3814, was identified as Aeromonas veronii using matrix-assisted laser desorption ionization–time of flight (MALDI-TOF) mass spectrometry (Bruker, Billerica, MA). SW3814 was grown (for DNA extraction and tests) overnight in tryptic soy broth (BD Bacto) at 35°C and stored in 25% glycerol at −80°C. Genomic DNA was obtained using a FastPrep homogenizer (MP Biomedicals) with 0.1-mm silica spheres, followed by the DNA extraction method described by Maniatis et al. (8). DNA was quantified with a Qubit fluorimeter (Life Technologies). An Illumina library was prepared with a Nextera DNA Flex library preparation kit, loaded into a 300-cycle high-output flow cell (2 × 150-bp paired-end reads), and run in a MiniSeq instrument with System Suite v2.0.0 (Illumina, San Diego, CA); 2,241,828 Illumina reads were obtained (reads with quality scores of >Q30, 91.1%). Illumina reads were quality filtered using fastp v0.23.1 (9). An Oxford Nanopore Technologies (ONT) (Oxford, UK) library was prepared using SQK-LSK109 and EXP-NBD196 kits, loaded into a FLO-MIN106D flow cell, and run in a MinION ONT device for 75 h. Base calling and quality filtering of ONT reads were conducted using Guppy for GPU v4.5.2; 18,373 ONT reads were obtained (mean size, 12,977 bp; minimum size, 1,000 bp; maximum size, 73,198 bp; reads with quality scores of >Q20, 63.2%). The genome was assembled using Unicycler v0.4.8-beta (10). The genome was annotated by the NCBI Prokaryotic Genome Annotation Pipeline (PGAP) with the best-placed reference protein set and GeneMarkS-2+ v5.3 (11–13). The plasmid copy number was obtained with Unicycler depth. Default parameters were used for all software. The Center for Genomic Epidemiology was used to annotate ARGs using ResFinder v2.1 (14). To determine the association of *bla*_VEB-3_ with MGEs, a BLASTn search was performed against the NCBI GenBank nucleotide database using the sequences of *bla*_VEB-3_ and IS*6100*. The top six matches with the greatest query coverage were used to create Fig. 1B.

SW3814 has a chromosome of 4,684,058 bp (GC content, 58.4%; Illumina coverage, 53.8×; ONT coverage, 45.8×), an IncA/C plasmid of 158,496 bp with 2.12 plasmid copies per chromosome (GC content, 51.8%), and a 1,739-bp plasmid with 16.92 copies per chromosome (GC content, 56.4%). ARGs found in the IncA/C plasmid were *aadA2*, *bla*_VEB-3_, *sul1*, *tetD*, *dfrA12*, *mphA*, and *strB* (Fig. 1A). The plasmid conferred resistance to five classes of antibiotics, classifying the strain as multidrug resistant (15). The *bla*_VEB-3_ gene was flanked by MGEs IS*6100* and IS*26* and a TnpF-like integrase (Fig. 1B). Similar genetic neighborhoods were found in other environmental and clinical bacteria (Fig. 1B and Table 1). The association of the VEB extended-spectrum β-lactamase (ESBL) with MGEs on an IncA/C plasmid highlights the potential for environmental *Aeromonas* strains to harbor and disseminate ARGs.

**TABLE 1:** Descriptions of isolates listed in Fig. 1B

Isolate	GenBank accession no.	Species	Country	Isolation source	Collection date(s)	Genetic location
A	GQ926879.1	Acinetobacter pittii	Taiwan	Blood of hospital patient	1999–2007^*a*^	Plasmid
B	CP018201.1	Aeromonas hydrophila	China	Water	2012	Chromosome
C	CP083462.1	Aeromonas veronii	USA	Lake in recreational park	2015	Plasmid
D	HM370390.1	Aeromonas caviae	France	Seine river	2009	Chromosome
E	CP006657.1	Klebsiella pneumonia	China	Blood of hospital patient	2010	Plasmid
F	HM370392.1	Aeromonas allosaccharophila	France	Seine river	2009	Plasmid
G	HM370391.1	Aeromonas veronii	France	Seine river	2009	Plasmid

a Clinical isolates were collected from three hospitals in Taiwan from 1999 to 2007.

### Data availability.

The genome sequence data for SW3814 have been deposited in NCBI GenBank under BioProject accession number PRJNA762937, BioSample accession number SAMN21418941, SRA accession number PRJNA762937, and GenBank accession numbers CP083461 (assembled chromosome), CP083462 (plasmid p158496), and CP083463 (plasmid p1739).
